# Obesity metabolomics signature in children: associations with metabolic abnormalities and potential biomarkers

**DOI:** 10.3389/fendo.2025.1671613

**Published:** 2025-09-25

**Authors:** Jiayi Wan, Shiyun Luo, Wanzhen Zhong, Guixian Tao, Jiaying Guo, Chunzi Zeng, Yujie Peng, Weiwei Zhang, Zhoubin Zhang, Jing Gu, Jie Huang, Yan Li

**Affiliations:** ^1^ School of Public Health, Sun Yat-sen University, Guangzhou, China; ^2^ Guangzhou Center for Disease Control and Prevention, Guangzhou Health Supervision Institute, Guangzhou, China

**Keywords:** obesity, metabolic abnormalities, metabolomics, oxidative stress, children

## Abstract

**Background:**

The global rise in childhood obesity has heightened its recognition as a major public health concern, with obesity being an independent risk factor for metabolic abnormalities. However, the metabolomics mechanisms linking pediatric obesity to metabolic abnormalities remain unclear.

**Methods:**

This case-control study utilized data from a 2023 cross-sectional survey of children aged 9–18 years in Guangzhou, China. A total of 246 participants were included, with 123 obese and 123 normal-weight participants matched for age and sex. Serum metabolomics profiling was performed via LC-MS. A dual machine learning approach combining penalized multivariable Least Absolute Shrinkage and Selection Operator (LASSO) regression and random forest with recursive feature elimination (RF-RFE) was employed to identify robust obesity-associated serum metabolites independent of metabolic abnormalities and logistic regression was employed to construct the obesity metabolomics signature (OB-MS) model. Multivariable logistic regression was used to assess the associations between the OB-MS and metabolic abnormalities and their components, including hyperglycemia, hypertension, hypertriglyceridemia, and reduced HDL-C.

**Results:**

Among 934 detected metabolites, 10 core metabolites were selected to construct the OB-MS, which showed high discriminative power, with an ROC-AUC of 0.986 in the testing set. Elevated OB-MS scores were significantly associated with increased risks of metabolic abnormalities, particularly hypertension and hypertriglyceridemia. Additionally, six key metabolites, including oxidative stress markers and dipeptides, were independently associated with metabolic abnormalities.

**Conclusions:**

This study established a pediatric obesity-specific metabolomics signature (OB-MS), implicating oxidative stress, protein catabolism, and glucocorticoid metabolism in obesity-related metabolic abnormalities. These finding illustrate the metabolic mechanisms underlying the relationship between childhood obesity and metabolic abnormalities and provide new scientific support for early and precise prevention of metabolic abnormalities in children. Further longitudinal studies and experimental validation are warranted to elucidate its biological mechanisms and clinical utility.

## 1 Introduction

Childhood obesity represents a major global health challenge, its prevalence having quadrupled since 1990 (1). According to the WHO, nearly 160 million young people were affected by obesity globally in 2022 (2). Beyond excessive adiposity, obesity constitutes a complex metabolic disorder that disrupts multiple physiological systems (e.g., lipid/amino acid metabolism, inflammation), particularly during childhood—critical periods for programming—leading to early metabolic abnormalities, including hyperglycemia (3, 4), hypertension (5) and dyslipidemia (6). Alarmingly, a substantial proportion of affected youth already exhibit clinically relevant metabolic abnormalities. Recent epidemiological data underscore this concern: approximately 18.0% of US adolescents are prediabetic (7), while in China, 9.0% and 10.0% of children suffer from hypertriglyceridemia and reduced high density lipoprotein cholesterol (HDL-C) (8), respectively. Globally, a 2020 Lancet review reported metabolic syndrome prevalence of 2.8% in children and 4.8% in adolescents, affecting millions (9). Critically, these early metabolic abnormalities represent a persistent long-term health threat (10), as longitudinal evidence confirms their tendency to track into adulthood significantly elevating the risk of subsequent metabolic disease morbidity and mortality (11). Consequently, elucidating the specific metabolic signatures inherent to childhood obesity and their links to metabolic abnormalities is imperative for developing targeted interventions aimed at mitigating the progression of metabolic abnormalities in this vulnerable population. Given the significant impact of obesity on metabolic abnormalities, it is essential to identify specific metabolic molecular mechanism associated with obesity in children and explore their relationships with metabolic abnormalities.

Metabolomics is the qualitative and quantitative analysis of endogenous small-molecule metabolites (<1500Da) and intermediates in biological systems (12). As metabolic activity represents the terminal level of biological regulation networks, metabolomics is regarded as a crucial bridge connecting genotypes and phenotypes (13). This technology offers considerable potential for discovering novel biomarkers, progression prediction, as well as determining the efficacy, safety, and dosage of therapeutic interventions (14). By identifying unique metabolic signatures associated with diseases, it facilitates earlier and more accurate diagnosis and disease subtyping than traditional methods (15).Furthermore, metabolomics guides personalized treatment strategies by monitoring dynamic metabolic responses to therapy, allowing for rapid adjustments to drug type and dosage to maximize efficacy and minimize adverse effects (12). This approach has proven invaluable in childhood obesity research, revealing obesity-specific metabolic disturbances, including elevated lysophosphatidylcholines (16), branched-chain amino acids (BCAAs) (17), and androgens (18). However, current metabolomics studies predominantly focus on group-level comparisons, leaving the integrated metabolic signature of obesity and its correlation with individual metabolic abnormalities largely unexplored.

To address these research gaps, we designed a case-control study including obesity and normal weight children/adolescent aged 9–18 years. A dual machine learning approach combining penalized multivariable Least Absolute Shrinkage and Selection Operator (LASSO) regression and random forest with recursive feature elimination (RF-RFE) were used to identify an obesity-associated metabolomics signature (OB-MS) independent of metabolic abnormality confounders. This OB-MS was served to discriminate obesity from normal-weight status and examined its associations with metabolic abnormalities and their components. This investigation aims to illustrate the underlying metabolic mechanisms by which obesity contributes to metabolic abnormalities and provide a scientific basis for precision prevention and early intervention of metabolic abnormalities in children.

## 2 Materials and methods

### 2.1 Study population

This case-control study utilized data from a cross-sectional survey conducted in 2023, which employed the multi-stage hierarchical cluster random sampling method to recruit children aged 9–18 years residing in Guangzhou, China. The specific sampling process has been detailed in our previous publication (19). A total of 116 participants were excluded because they had incomplete baseline data regarding anthropometric or biochemical measurements and 1449 participants were finally enrolled. All participants completed the questionnaire, anthropometric and biochemical measurements.

In this case-control study, childhood weight status was classified as normal weight (<85th) and obesity (>95th) by the age- and sex-specific BMI cutoff points for screening among children aged 2–18 years recommended by the National Health and Family Planning Commission of China in 2018 (20). Therefore, 123 participants with obesity as cases and 123 participants with normal weight (NW) as controls, randomly matched for similar age (± 3 years old) and sex, to perform serum untargeted metabolomics detection. The screening process of the research participants is shown in **Supplementary Figure 2**.

The present study adhered to the Declaration of Helsinki and received approval from the Ethics Committee of Guangzhou Center for Disease Control and Prevention (ethics number GZCDC-ECHR-2021P0019 and GZCDC-ECHR-2022P0038). Informed consent forms were signed by all participants and their legal guardians.

### 2.2 Definitions of metabolic abnormalities

Metabolic abnormalities were defined as the presence of a metabolically unhealthy state (21), based on the following four metabolic components: (1) Hyperglycemia: fasting plasma glucose (FPG)>5.6mmol/L (22); (2) Hypertension: systolic blood pressure(SBP) and/or diastolic blood pressure(DBP) >90th percentile for age- and sex-specific group (23); (3)Hypertriglyceridemia: triglyceride(TG) >1.70mmol/L; (4) Reduced HDL-C: HDL-C ≤ 1.03mmol/L. Participants who met one or more of these criteria were defined as metabolic abnormalities.

### 2.3 Anthropometric and biochemical measurements

Anthropometric measurements were conducted by trained staff using standardized procedures according to the technical standards for student physical examinations (24). Height was measured using a metallic column stature meter with a 0.1 cm precision. Weight was measured, in light clothes, using a set of standard calibrated electronic scales with a precision of 0.1 kg. Waist circumstance (WC) was measured using a glass fiber ruler with a precision of 0.1 cm. The body mass index (BMI) was calculated as weight divided by the square of height (kg/m^2^). Blood pressures (SBP and DBP) were measured using automatic electronic sphygmomanometers with a 1 mmHg precision. These blood pressure measurements were taken twice after several minutes of rest in a sitting position and the mean values were recorded as the final BP values.

Venous blood samples for biochemical analyses were collected from participants after an overnight fast. The blood samples were immediately centrifuged to separate the serum, which was stored at −80°C. TG was measured by the oxidase method. HDL-C was measured by the direct method. FPG was measured by the hexokinase method.

### 2.4 Serum metabolomics detection

The stored serum samples were thawed on ice and vortexed for 10 s prior to analysis. 50 μL of each sample and 300 μL of extraction solution, which included internal standards and had an ACN to Methanol ratio of 1:4 (v/v), were added into a 2mL microcentrifuge tube and vortexed for 3 min. After centrifugation (4 °C, 10 min, 12000 rpm), 200 μL of the supernatant was collected and placed in -20 °Cfor 30 min, and then centrifuged at 12000 rpm for 3 min (4 °C). A 180 μL aliquots of supernatant were transferred for LC-MS analysis. The specific program for LC-MS analysis was attached to the **Supplementary Material**.

### 2.5 Covariates

The covariates in this study included age, sex, parental education level, smoking status, moderate-to-high-intensity exercise status, screen time status, dietary diversity status. Parent’s education level, including both father and mother, were divided into two categories as high school or below and junior college or above. Smoking was classified as smokers and non-smokers. Moderate-to-high-intensity exercise status was categorized as ≥1h/day and <1h/day (25). Screen time was classified as >2h/day and ≤2h/day (25). Dietary diversity status was divided into two levels as high-level DDS and low-level DDS base on the median of dietary diversity scores (DDS). According to the 2016 Dietary Guidelines for the Chinese, DDS was calculated based on nine major food groups: cereals; white tubers and roots; legumes, legume products, nuts, and seeds; vegetables and vegetable products; fruits; meat; eggs; fish and fish products; and milk and milk products. One point was added to the DDS for each food group consumed in the past month, with no double-counting within the same food group. The DDS ranged from 1 to 9 (26, 27).

### 2.6 Data Analysis

All metabolite data were log-transformed to reduce skewness and then mean-normalized prior to analysis. Baseline data were shown as median [lower interquartile range (Q1), up interquartile range (Q3)] or n (%) for continuous variables and categorical variables. Baseline characteristics were compared using Mann-Whitney U tests for continuous variables and chi-square for categorical variables. To account for multiple testing, the false discovery rate (FDR) was calculated using the Benjamini-Hochberg (BH) method and statistically significant was considered as FDR<0.05.

A total of 246 samples were randomly split into a training set (70%, n=172) and a testing set (30%, n=74) to develop and evaluate the prediction model related to OB-MS. We implemented a dual machine learning strategy combining penalized multivariable LASSO regression and RF-RFE, both employing 10-fold cross-validation to identify obesity-associated metabolites independent of metabolic abnormalities. In the LASSO approach, we fixed metabolic abnormalities and their components, including hyperglycemia, hypertension, hypertriglyceridemia, and reduced HDL-C, as non-penalized covariates (λ=0) while applying L1 regularization to other metabolites, with the optimal λ determined by minimizing the cross-validated mean squared error. For RF-RFE, we similarly retained these metabolic covariates in all splits while recursively eliminating other metabolites features based on importance rankings through 10-fold cross-validation, ultimately selecting the candidate metabolite subset by maximizing the Kappa statistic. By using consistent cross-validation and rigorously controlling for metabolic confounders, this parallel implementation of regularized regression and ensemble-based feature selection enhanced the reliability of identified obesity-specific metabolomics signature.

The final OB-MS model was then constructed using logistic regression, with obesity status as the dependent variable and the metabolites jointly selected by dual machine learning strategy as the independent variables. Subsequently, the model was applied to the testing set to assess effectiveness and accuracy by receiver-operator area-under-the-curve (ROC-AUC), accuracy, recall, precision and F1 score. Finally, we assigned the coefficients obtained from the final OB-MS logistic regression model as weights to the selected metabolites, and then calculated the OB-MS score as the sum of multiples of these weights and their corresponding values.

The OB-MS score was transformed to Z-score (mean=0, SD = 1) and categorized into quartiles. To examine the relationship between the OB-MS and metabolic abnormalities and their components, including hyperglycemia, hypertension, hypertriglyceridemia, and reduced HDLC, we conducted two multivariable logistic regression. The first model was adjusted for age and sex, and the second model was further adjusted for parental education level, smoking status, moderate-to-high-intensity exercise, screen time, dietary diversity. Additionally, we explored the association between individual metabolites and metabolic abnormalities and their components by multivariate logistic regression. Multiple testing corrections were done using the Benjamini-Hochberg method and FDR<0.05 was considered significant.

All modeling and statistical analysis were performed in R4.1.1. The biological functions and metabolic pathways of metabolites were obtained from KEGG (https://www.genome.jp/kegg/) and HMDB (https://hmdb.ca/).

## 3 Results

### 3.1 Baseline characteristics

A total of 246 participants were enrolled in this study, with a median age of 13.6 years. As shown in **Table 1**, no significant differences were observed between two groups in terms of the demographic characteristics and lifestyle characteristics, including age, sex, parental education level, smoking, moderate-to-high-intensity exercise, screen time, dietary diversity (FDR>0.05). Compared with NW, obesity had higher levels of BMI, WC, SBP, TG and lower levels of HDL-C (FDR<0.05).

**Table 1:** General characteristics of the participants.

Variable	Total (*n* = 246)	NW (*n* = 123)	Obesity (*n* = 123)	*Z/χ* ^2^	*P*	*FDR*
Age (year)	13.6 (11.0, 14.3)	13.7 (10.9, 14.1)	13.4 (11.2, 14.9)	-1.066	0.286	0.349
Sex				0.000	1.000	1.000
Boys	172 (69.9)	86 (69.9)	86 (69.9)			
Girls	74 (30.1)	37 (30.1)	37 (30.1)			
Education of father				2.156	0.124	0.234
High school or below	184 (74.8)	97 (78.9)	87 (70.7)			
Junior college or above	62 (25.2)	26 (21.1)	36 (29.3)			
Education of mother				2.482	0.115	0.234
High school or below	179 (72.8)	95 (77.2)	84 (68.3)			
Junior college or above	67 (27.2)	28 (22.8)	39 (31.7)			
Lifestyle characteristics						
Smoking				1.038	0.308	0.349
Smokers	9 (3.7)	6 (4.9)	3 (2.4)			
Non-smokers	237 (96.3)	117 (95.1)	120 (97.6)			
Moderate-to-high-intensity exercise				1.491	0.222	0.317
<1h/day	165 (67.1)	87 (70.7)	78 (63.4)			
≥1h/day	81 (32.9)	36 (29.3)	45 (36.6)			
Screen time				1.480	0.224	0.317
≤2h/day	190 (77.2)	91 (74.0)	99 (80.5)			
>2h/day	56 (22.8)	32 (26.0)	24 (19.5)			
Dietary diversity				1.056	0.304	0.349
High-level DDS	108 (43.9)	58 (47.2)	50 (40.7)			
Low-level DDS	138 (56.1)	65 (52.8)	73 (59.3)			
Anthropometric and biochemical measurements						
BMI (kg/m^2^)	22.1 (18.2, 27.7)	18.2 (16.8, 19.1)	27.7 (25.9, 29.3)	-13.548	**<0.001**	**<0.001**
WC (cm)	70.0 (61.8, 84.0)	62.0 (58.4, 65.5)	84.0 (76.7, 90.0)	-13.070	**<0.001**	**<0.001**
SBP (mmHg)	112.0 (105.0, 115.0)	112.0 (105.0, 112.0)	113.0 (106.0, 120.0)	-4.815	**<0.001**	**<0.001**
DBP (mmHg)	70.0 (67.0, 74.3)	69.0 (67.0, 72.0)	71.0 (67.0, 75.0)	-1.394	0.163	0.277
FPG (mmol/L)	5.1 (4.8, 5.3)	5.1 (4.8, 5.3)	5.0 (4.7, 5.3)	-2.138	**0.033**	0.080
TG (mmol/L)	0.9 (0.8, 1.3)	0.9 (0.7, 1.1)	1.0 (0.8, 1.5)	-2.697	**0.007**	**0.023**
HDL-C (mmol/L)	1.2 (1.1, 1.4)	1.3 (1.1, 1.5)	1.2 (1.1, 1.3)	-3.141	**0.002**	**0.009**

Data are presented as median (IQR) or n (%). *P* values derived from the Mann-Whitney U Test for continuous data and the Chi-square test for categorical data, respectively. Bold *p* values were indicated “<0.05”. FDR, false discovery rate; BMI, body mass index; WC, waist circumference; SBP, systolic blood pressure; DBP, diastolic blood pressure; FPG, fasting plasma glucose; TG, triglycerides; HDL-C, high density lipoprotein cholesterol.

### 3.2 Identify metabolomics signature of obesity by machine learning approaches

**Figure 1:**
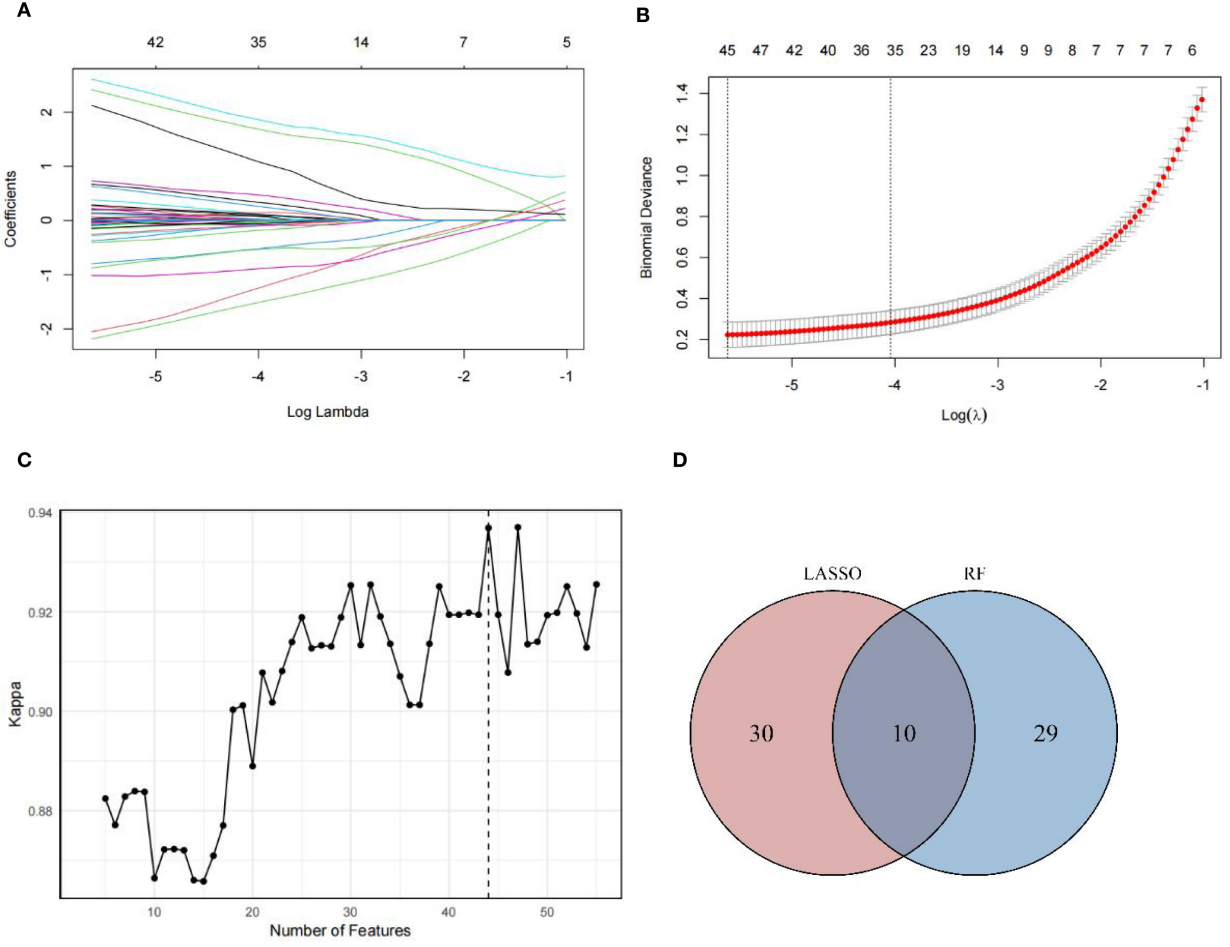
Metabolites screening results. **(A)** Plots for the LASSO regression coefficients across various penalty parameter *λ*. As the value of *λ* increased, the regularization strength increased and more variable coefficients were compressed to zero. **(B)** Cross-validation plots for the penalty parameter *λ*. The vertical dashed line on the left indicated the value of the log(*λ*) when the error of the model was minimized. Excluding 5 fixed covariates, 40 metabolites were selected as candidate metabolites corresponding to the minimum *λ* (0.004). **(C)** Kappa values across number of features in RF model. As the number of features increased, the Kappa value increased, indicating an improvement in model performance. In optimization model, 44 features were selected, including 5 fixed covariates and 39 metabolites, achieving the Kappa value of 0.937. **(D)** Overlap of candidate metabolites selected by LASSO and RF-RFE models. The intersection of metabolites screened by LASSO and RF-RFE, with 10 shared metabolites were identified as robust candidates for final OB-MS model construction.

A total of 934 metabolites were detected in serum samples. To identify robust obesity-associated metabolites independent of metabolic abnormalities, we employed penalized multivariable LASSO regression and RF-RFE, both implemented with 10-fold cross-validation. In the LASSO regression analysis, we incorporated metabolic abnormalities and their components (hyperglycemia, hypertension, hypertriglyceridemia and reduced HDL-C) as mandatory covariates by fixing their penalty coefficients at λ=0, while applying L1 regularization to all other metabolites for feature selection. Through 10-fold cross-validation, we adopted the minimum λ (λ=0.004) to fit the final LASSO logistic regression model (**Figure 1A**). This model identified 40 metabolites with non-zero coefficients (**Figure 1B**) after adjusting for above metabolic confounders. In the RF-RFE model, we similarly retained these metabolic covariates in all splits while recursively eliminating other metabolites features based on importance rankings through 10-fold cross-validation. Feature selection achieved peak performance (maximum Kappa=0.937) when retaining 44 features, comprising 39 selected metabolites and the 5 fixed covariates (**Figure 1C**). The coefficients of candidate metabolites in the LASSO model and the importance ranking of the candidate metabolites in the optimal RF-RFE model were shown in **Supplementary Figures 3** and **Supplementary Figure 4**. The intersection of two methods further revealed 10 overlapping metabolites, underscoring their robust association with obesity independent of metabolic abnormalities (**Figure 1D**).

### 3.3 The construction of the OB-MS

**Figure 2:**
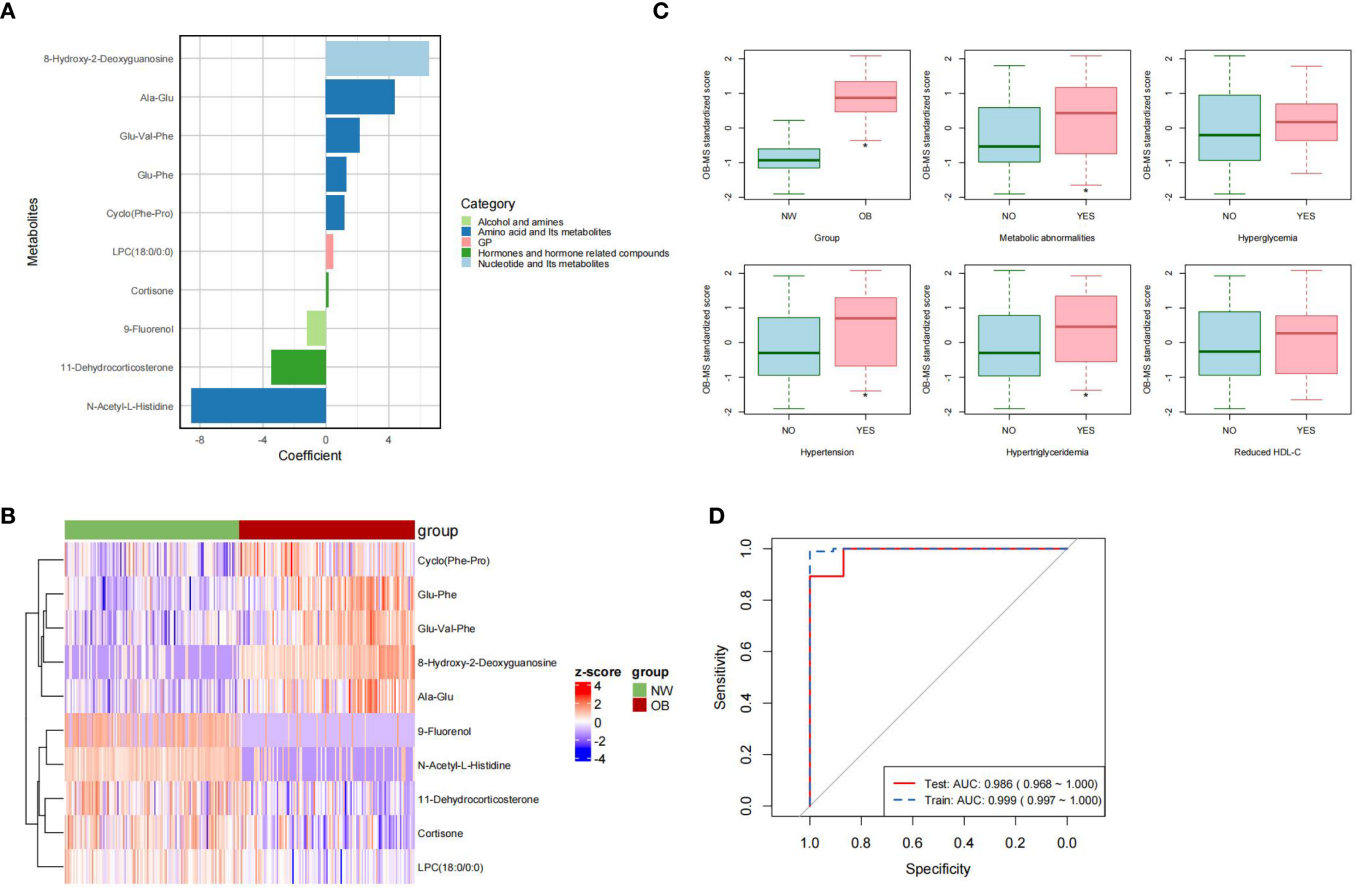
Characteristic of the OB-MS. **(A)** The regression coefficients in the OB-MS model. The metabolites were ranked from the highest to the lowest coefficient value and were color-code according to their class, respectively. **(B)** The cluster heatmap of the OB-MS across normal weight and obesity. The cells were filled with Z-scores derived from mean-normalization of the relative abundance. Red cells indicated values above the average and the blue indicated values below the average. **(C)** Distribution of the OB-MS scores across metabolic abnormalities subgroups. The asterisk (*) indicates statistically significant differences between groups by *t* test. (*p* < 0.05). **(D)** Receiver-operator curve (ROC) for the final OB-MS model. The red solid line indicated the prediction performance in the training set, while the blue dashed line indicated the performance in the testing set based on the final OB-MS model.

The OB-MS model was developed using logistic regression analysis, incorporating 10 stable metabolites as independent variables and obesity status as the dependent variable. The regression coefficients of each metabolite were calculated. Regression coefficients were calculated for each metabolite, with 8-hydroxy-2-deoxyguanosine exhibiting the highest positive coefficient and N-acetyl-L-histidine showing the most negative coefficient (**Figure 2A**). Significant differences were identified in the mean normalized distribution of these obesity-association metabolites across NW and obesity groups (**Figure 2B**). By assigning the regression coefficients as weights to metabolites, we calculated the OB-MS score as the sum of multiples of these weights and their corresponding values. The OB-MS score was significantly higher in obesity individuals compared to NW controls (*p* < 0.05). Additionally, elevated OB-MS scores were consistently observed in subgroups with metabolic abnormalities, hypertension and hypertriglyceridemia (all *p* < 0.05, **Figure 2C**).

To evaluate the predictive performance of the OB-MS model, we examined its classification accuracy in the test set. In the testing set (*n* = 74), the model correctly classified 66 subjects, achieving the accuracy of 0.892, recall of 0.929, precision of 0.813 and F1 score of 0.867. To further evaluate the performance of the model, the receiver-operator curves (ROC) were constructed for both the training set and the testing set. The ROC-AUC in the training set was 0.999, with a 95% confidence interval (*CI*) ranging from 0.997 to 1.000, while in the testing set, the ROC-AUC was 0.986, with a 95% *CI* ranging from 0.968 to 1.000 (**Figure 2D**). These results underscored the robustness and high discriminative power of the OB-MS in distinguishing NW and obesity.

### 3.4 Associations of the OB-MS scores with metabolic abnormalities and their components

In this study, we employed multivariable logistic regression analysis to explore the associations between the OB-MS scores and metabolic abnormalities and their components, including hyperglycemia, hypertension, hypertriglyceridemia and reduced HDL-C. The results revealed that after adjusting for age, sex, parental education level, smoking status, moderate-to-high-intensity exercise, screen time and dietary diversity, compared to the Q1 group (the lowest quartile), children in the Q4 group (the highest quartile) of the OB-MS scores showed significantly higher risks for metabolic abnormalities (*OR* = 2.806, 95% *CI*: 1.290 ~ 6.013), hypertension (*OR* = 2.449, 95% *CI*: 1.034 ~ 5.803) and hypertriglyceridemia (*OR* = 9.088, 95% *CI* = 1.953 ~ 42.284). Similarly, after adjustments of above covariates, per 1-SD increased in the OB-MS score was significantly associated with an increased risk of metabolic abnormalities (*OR* = 1.797, 95% *CI*: 1.354 ~ 2.386), hypertension (*OR* = 1.621, 95% *CI*: 1.167 ~ 2.252) and hypertriglyceridemia (*OR* = 1.947, 95% *CI*: 1.290 ~ 2.938), as shown in **Table 2**. Although significant differences were observed in certain groups for hyperglycemia and reduced HDL-C, overall trend tests and linear relationship analyses did not reveal a consistent trend with the OB-MS scores.

**Table 2:** Associations of the OB-MS score with Metabolic abnormalities and their components.

Outcome	Case, *n* (%)	Q2		Q3		Q4		*P*-trend	Per 1-SD increased	
		*OR* (95% *CI*)	*P*	*OR* (95% *CI*)	*P*	*OR* (95% *CI*)	*P*		*OR* (95% *CI*)	*P*
Metabolic abnormalities	126 (51.2)									
Model1		0.624 (0.294 – 1.328)	0.221	3.100 (1.461 – 6.577)	**0.003**	2.953 (1.401 – 6.224)	**0.004**	**<0.001**	1.817 (1.382 – 2.391)	**<0.001**
Model2		0.573 (0.263 – 1.251)	0.162	3.099 (1.427 – 6.730)	**0.004**	2.806 (1.290 – 6.103)	**0.009**	**<0.001**	1.797 (1.354 – 2.386)	**<0.001**
Hyperglycemia	21 (8.5)									
Model1		1.333 (0.282 – 6.300)	0.717	5.467 (1.421 – 21.032)	**0.014**	0.557 (0.087 – 3.577)	0.537	0.697	1.051 (0.671 – 1.646)	0.829
Model2		1.347 (0.277 – 6.557)	0.712	5.690 (1.421 – 22.786)	**0.014**	0.610 (0.091 – 4.082)	0.611	0.597	1.084 (0.681 – 1.725)	0.734
Hypertension	52 (21.1)									
Model1		0.406 (0.132 – 1.244)	0.115	1.137 (0.464 – 2.787)	0.779	2.580 (1.119 – 5.944)	**0.026**	**0.006**	1.672 (1.211 – 2.307)	**0.002**
Model2		0.387 (0.124 – 1.209)	0.102	1.056 (0.421 – 2.649)	0.908	2.449 (1.034 – 5.803)	**0.042**	**0.010**	1.621 (1.167 – 2.252)	**0.004**
Hypertriglyceridemia	32 (13.0)									
Model1		3.471 (0.668 – 18.044)	0.139	6.634 (1.372 – 32.079)	**0.019**	9.088 (1.953 – 42.284)	**0.005**	**0.001**	2.026 (1.355 – 3.031)	**0.001**
Model2		3.258 (0.617 – 17.207)	0.164	6.319 (1.287 – 32.028)	**0.023**	8.199 (1.726 – 38.952)	**0.008**	**0.002**	1.947 (1.290 – 2.938)	**0.002**
Reduced HDL-C	50 (20.3)									
Model1		1.063 (0.405 – 2.792)	0.901	2.371 (0.977 – 5.759)	0.057	1.275 (0.502 – 3.241)	0.610	0.294	1.130 (0.829 – 1.541)	0.440
Model2		1.036 (0.389 – 2.755)	0.944	2.553 (1.031 – 6.322)	**0.043**	1.247 (0.479 – 3.243)	0.651	0.295	1.132 (0.823 – 1.556)	0.446

OR, odds ratios; CI, confidence interval; HDL-C, high density lipoprotein cholesterol. Model 1 was adjusted for age and sex. Model 2 was adjusted for age, sex, parental education level, smoking status, moderate-to-high-intensity exercise, screen time and dietary diversity. Bold *p*-values were indicated “<0.05”.

### 3.5 Associations of individual obesity-association metabolites with metabolic abnormalities and their components

**Figure 3:**
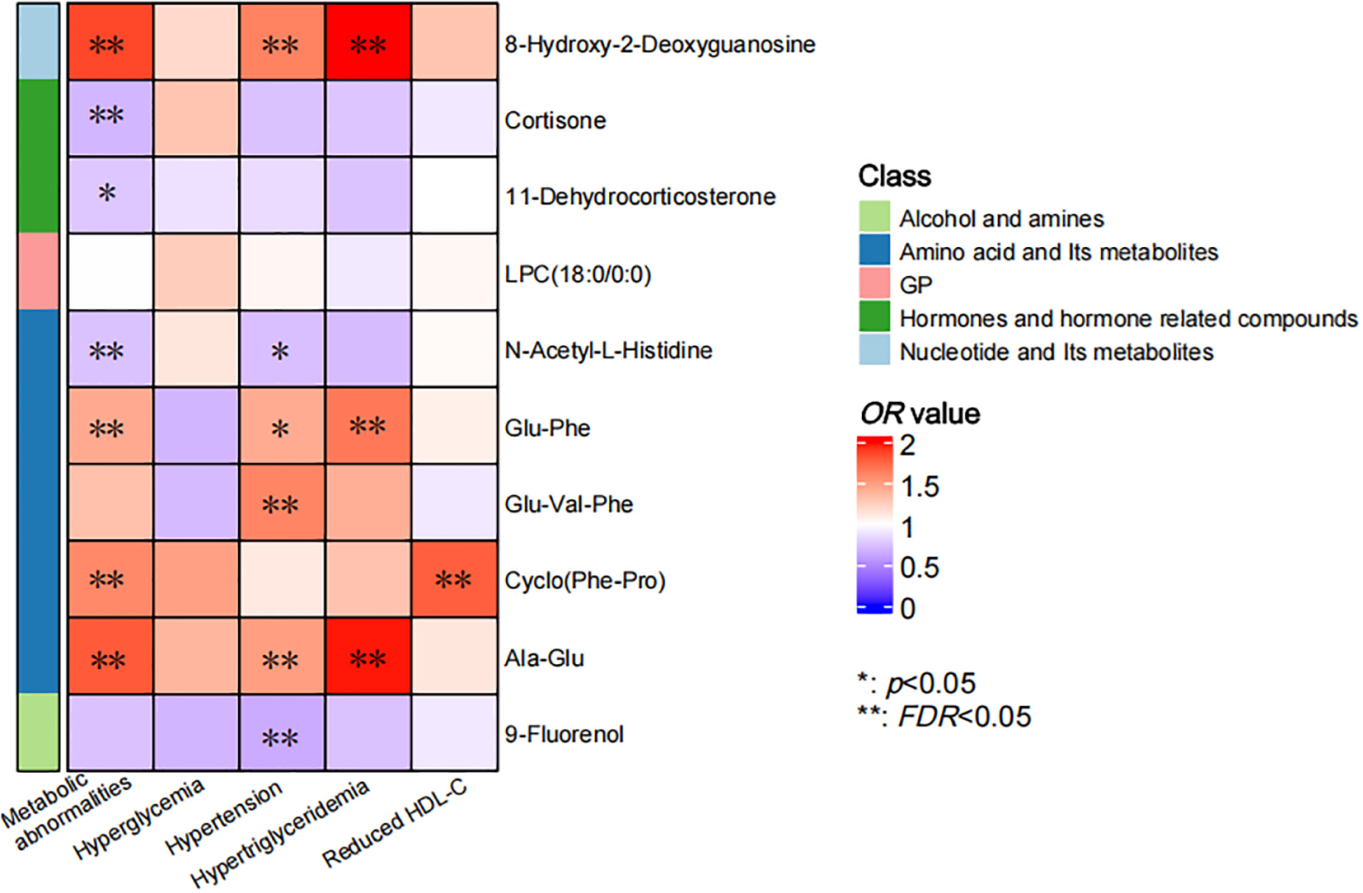
Correlation of individual metabolites and metabolic abnormalities and their components. The color bar indicated the *OR* value, with the red representing values higher than 1 and the blue representing values lower than 1. The asterisks denoted statistical significance, with “*” for *p* values less than 0.05 and “**” for FDR less than 0.05.

The correlations between individual metabolites and metabolic abnormalities and their components were assessed using multivariate logistic regression (**Figure 3**). After adjustment for covariates in above Model2, 6 metabolites showed significant independent associations with metabolic abnormalities (*FDR* < 0.05). The oxidative stress marker 8-hydroxy-2-deoxyguanosine showed the strongest positive association (*OR* = 1.871, 95% *CI*: 1.404 ~ 2.495), followed by Ala-Glu, Cyclo (Phe-Pro) and Glu-Phe. Conversely, N-acetyl-L-histidine (*OR* = 0.737, 95% *CI*: 0.566 ~ 0.960) and cortisone exhibited significant negative associations. Furthermore, we identified specific potential biomarker metabolites independently associated with hypertension, hypertriglyceridemia and reduced HDL-C. Specifically, 8-hydroxy-2-deoxyguanosine, Glu-Val-Phe, Ala-Glu and 9-Fluorenol were significantly associated with hypertension. 8-hydroxy-2-deoxyguanosine, Glu-Phe and Ala-Glu were significantly associated with hypertriglyceridemia and Cyclo (Phe-Pro) was significantly associated with reduced HDL-C (all *FDR* < 0.05). Collectively, these metabolites may serve as potential biomarkers for metabolic abnormalities and their specific components.

## 4 Discussion

In this case-control study, we used a dual machine learning strategy to identify core serum metabolites associated with obesity but independent of metabolic abnormalities. The regularized LASSO regression model selected 40 candidate metabolites, while RF-RFE selected 39 candidate metabolites, with 10 overlapping metabolites robustly linked to obesity after adjusting for metabolic confounders. These 10 metabolites were integrated into the OB-MS, which demonstrated high discriminative accuracy in distinguishing obesity individuals from normal-weight controls. Furthermore, we found significant associations between OB-MS and metabolic abnormalities, particularly hypertension and hypertriglyceridemia, highlighting its potential as both a diagnostic tool and a biomarker for metabolic abnormalities.

The OB-MS, derived from 10 obesity-associated metabolites, reflects key pathophysiological pathways linking obesity to metabolic abnormalities. Among these metabolites highly associated with childhood obesity, 8-OHdG exhibits the strongest positive correlation, while N-Acetyl-L-Histidine exhibits the strongest negative correlation. As a widely recognized marker of oxidative stress, 8-OHdG is formed through the hydroxylation of the guanine base in DNA by reactive oxygen species (ROS), particularly at the C8 position (28). This oxidative modification primarily occurs when excessive ROS production overwhelms cellular antioxidant defenses, leading to DNA damage and subsequent excision repair that releases 8-OHdG into circulation (29). The significantly elevated levels of 8-OHdG observed in our study strongly implicate oxidative stress as a crucial pathological mechanism in obesity (30, 31).The negative association of N-Acetyl-L-Histidine suggests potential neuroprotective mechanisms that may be compromised in obesity children, which is rarely reported in adult cohorts and need to be further explored on the neural mechanisms (32, 33).

Cortisone, the inactive precursor to cortisol, and 11-dehydrocorticosterone, the corticosterone counterpart, are interconverted with their active forms through tissue-specific 11 β-hydroxysteroid dehydrogenase (11 β-HSD) enzymes (34, 35). Under physiological conditions, 11β-HSD1 activates cortisone to cortisol (and 11-dehydrocorticosterone to corticosterone), while 11β-HSD2 mediates the reverse inactivation (36), which allows for maintaining a balance of their activities (37). However, in obesity, adipose 11β-HSD1 is likely upregulated, increasing local cortisol levels while depleting systemic glucocorticoid precursors (38, 39). Concurrently, chronic low-grade inflammation characteristic of obesity may suppress the hypothalamic-pituitary-adrenal axis through negative feedback mechanisms, reducing adrenal production of both precursors and active glucocorticoids (40–42). This creates a paradoxical state of tissue-specific glucocorticoid excess alongside systemic deficiency, consistent with our findings, reflecting the complex dysregulation of glucocorticoid metabolism in obesity. Interestingly, while 11-dehydrocorticosterone is classically considered a rodent biomarker, its detectable levels in humans may reflect conserved metabolic dysregulation (43, 44).

Our findings demonstrate significant alterations in protein catabolism among obesity children, characterized by the accumulation of specific dipeptides (e.g., Glu-Phe, Ala-Glu). However, current research remains divided regarding the impact of obesity on protein breakdown and synthesis. Some studies suggest that excess energy intake in obesity is predominantly channeled into lipogenesis without significantly altering tissue protein turnover rates (45, 46). In contrast, other studies have shown that obesity-associated proinflammatory cytokines activate the ubiquitin-proteasome pathway to enhance protein degradation, while oxidative stress-induced protein damage further modulates the production and metabolic homeostasis of peptides (47, 48). The specific mechanisms underlying obesity-mediated protein metabolic dysregulation remain incompletely understood, warranting more comprehensive investigations in future research.

Our metabolomics analysis demonstrates that OB-MS scores are significantly associated with oxidative stress and inflammatory responses. Elevated OB-MS scores showed independent associations with increased risks of metabolic abnormalities, hypertension, and hypertriglyceridemia in children, after adjusting for age, sex, and lifestyle factors. These findings suggest that obesity-related metabolic disturbances, characterized by oxidative stress, may underlie the development of metabolic abnormalities. Importantly, these alterations may represent early mechanistic links between childhood obesity and subsequent cardiometabolic disease progression. Obesity-associated chronic inflammation can suppresses lipoprotein lipase activity, further impairing peripheral tissue clearance of triglycerides (49). Excessive adipose tissue accumulation leads to hypertrophy and dysfunction of adipocytes, which secrete large amounts of pro-inflammatory cytokines, such as TNF-α, and free fatty acids (50). These factors activate NADPH oxidase, producing ROS that damage vascular endothelial cells (51), reducing nitric oxide (NO) bioavailability and impairing vascular dilation. Additionally, ROS activation of the renin-angiotensin system (RAS) promotes vasoconstriction and sodium retention, contributing to hypertension (52, 53). However, our study did not identify significant associations between OB-MS scores and hyperglycemia and Reduced HDL-C in children, which shows inconsistency with some previous findings (54–56). These results may be related to our sample size limitations and the inherent constraints of cross-sectional studies in capturing the dynamic progression of metabolic abnormalities, which suggest the necessity of incorporating more refined metabolic subtyping and dynamic indicators for comprehensive metabolic risk assessment in obese pediatric populations.

Given the crucial role of oxidative stress in obesity-related metabolic abnormalities, the importance of clinical anti-inflammatory management in pediatric obesity cannot be overstated. Some healthy diets that have demonstrated the efficacy and benefits of healthy diets in maintaining low-grade inflammation are suggested to apply in pediatric obesity populations (57), such as traditional mediterranean diet (58, 59) and dash diet (60, 61). These beneficial dietary patterns are characterized by higher proportions of whole grains, fruits and vegetables, as well as lower consumption frequencies of red meat and its products. Besides, increasing the intake of specific foods, such as omega-3 fatty acids (62) and polyphenol (63, 64) may provide additional anti-inflammatory benefits. Meanwhile, we recommend dynamic monitoring of inflammatory biomarkers like 8-OHdG to identify potential high-risk populations with early metabolic abnormalities and objectively evaluate intervention efficacy of obesity interventions, thereby enabling personalized treatment adjustments. These recommendations allow healthcare providers to tailor intervention strategies more precisely, thereby reducing the risk of metabolic abnormalities in pediatric obesity patients and ultimately improving their long-term health outcomes.

This study employed a dual machine learning strategy to identify 10 core metabolites independently associated with obesity after adjusting for confounding metabolic abnormalities, and constructed a highly discriminative obesity metabolomics signature (OB-MS). Compared to previous studies, our approach enhanced the robustness of metabolite selection (65, 66) and identified obesity-specific metabolites independent of metabolic abnormalities (18, 67). The OB-MS effectively distinguished obesity individuals from normal-weight controls and demonstrated independent associations with hypertension and hypertriglyceridemia, suggesting its potential as an early biomarker for obesity-related metabolic disorders. Furthermore, we identified significant metabolic features in childhood obesity, e.g., 8-OHdG, providing novel insights into the pathophysiology of pediatric obesity. These findings contribute to a deeper understanding of the relationship of pediatric obesity and metabolic abnormalities, and also reveal the clinical potential of targeted metabolomics profiling to identify high risk subgroups and guide precision interventions.

Several limitations of this study should be acknowledged. First, as with most prior metabolomics studies, our case-control design captures a metabolic snapshot but lacks longitudinal data to track dynamic changes over time, making it difficult to establish clear causal relationships. Second, our untargeted metabolomics approach provides comprehensive metabolic profiling but remains limited by its single-omics design. The absence of incorporating other omics data may overlook important biological interactions and confounding factors influencing metabolic abnormalities. Third, the lack of external validation cohorts representing diverse ethnic and geographic populations restrict our ability to assess the generalizability of the findings across diverse populations. In addition to the limitations of this study, the widespread application of metabolomics findings to clinical practice still faces challenges. On the one hand, the inconsistency between processes and laboratory instruments makes it difficult to establish a unified metabolite reference database, which in turn affects reproducibility and comparability. In addition, the high operating costs, complexity of biomarker validation, and lengthy regulatory approval processes all limit the clinical application of newly discovered biomarkers.

Despite these limitations, our findings open several promising avenues for future research. Longitudinal cohort studies are essential to establish the OB-MS as a predictive biomarker for the development of metabolic abnormalities and to track its trajectory from childhood into adulthood. Concurrently, experimental studies are required to elucidate the mechanisms by which these core metabolites (e.g., 8-OHdG) mediate the development of metabolic abnormalities. Moreover, intervention studies should explore whether lifestyle modifications or pharmacological therapies can effectively modulate the OB-MS and thereby improve metabolic health outcomes. From a translational perspective, future work should address the challenges in metabolomics, such as protocol standardization and cost reduction, to facilitate the clinical application of these biomarkers for early risk stratification and personalized prevention strategies.

## 5 Conclusion

By rigorous machine learning approach and control for metabolic confounders, this study established the pediatric obesity-specific metabolomics signature (OB-MS), highlighting the critical roles of oxidative stress, protein catabolism and glucocorticoid metabolism in childhood obesity. The OB-MS not only serves as a novel diagnostic tool but also suggests potential early intervention targets through its associations with metabolic abnormalities. Further longitudinal studies and experimental validation are warranted to elucidate its biological mechanisms and clinical utility.

## Data Availability

The original contributions presented in the study are included in the article/**Supplementary Material**. Further inquiries can be directed to the corresponding authors.

## References

[1] Lister NB Baur LA Felix JF Hill AJ Marcus C Reinehr T. Child and adolescent obesity. Nat Rev Dis Primers. (2023) 9:1–19. doi: 10.1038/s41572-023-00435-4, PMID: 37202378

[2] NCD Risk Factor Collaboration (NCD-RisC). Worldwide trends in underweight and obesity from 1990 to 2022: a pooled analysis of 3663 population-representative studies with 222 million children, adolescents, and adults. Lancet. (2024) 403:1027–50. doi: 10.1016/s0140-6736(23)02750-2, PMID: 38432237 PMC7615769

[3] Cucuzzella M Bailes J Favret J Paddu N Bradley AB. Beyond obesity and overweight: the clinical assessment and treatment of excess body fat in children : part 1 - insulin resistance as the root cause of pediatric obesity. Curr Obes Rep. (2024) 13:276–85. doi: 10.1007/s13679-024-00565-0, PMID: 38709471

[4] Landgraf K Rockstroh D Wagner IV Weise S Tauscher R Schwartze JT. Evidence of early alterations in adipose tissue biology and function and its association with obesity-related inflammation and insulin resistance in children. Diabetes. (2015) 64:1249–61. doi: 10.2337/db14-0744, PMID: 25392242

[5] de Simone G Mancusi C Hanssen H Genovesi S Lurbe E Parati G. Hypertension in children and adolescents. Eur Heart J. (2022) 43:3290–301. doi: 10.1093/eurheartj/ehac328, PMID: 35896123

[6] Zeljkovic A Vekic J Stefanovic A. Obesity and dyslipidemia in early life: Impact on cardiometabolic risk. Metabolism. (2024) 156:155919. doi: 10.1016/j.metabol.2024.155919, PMID: 38653373

[7] Andes LJ Cheng YJ Rolka DB Gregg EW Imperatore G. Prevalence of prediabetes among adolescents and young adults in the United States, 2005-2016. JAMA Pediatr. (2020) 174:e194498. doi: 10.1001/jamapediatrics.2019.4498, PMID: 31790544 PMC6902249

[8] Zhou Z Jia Y Yan H Xu J Wen J Wang S. Meta-analysis of the prevalence of dyslipidemia among Chinese children and adolescents. Chin Gen Pract. (2014) 27:2145–54. doi: 10.12114/j.issn.1007-9572.2023.0806

[9] Noubiap JJ Nansseu JR Lontchi-Yimagou E Nkeck JR Nyaga UF Ngouo AT. Global, regional, and country estimates of metabolic syndrome burden in children and adolescents in 2020: a systematic review and modelling analysis. Lancet Child Adolesc Health. (2022) 6:158–70. doi: 10.1016/s2352-4642(21)00374-6, PMID: 35051409

[10] Juonala M Magnussen CG Berenson GS Venn A Burns TL Sabin MA. Childhood adiposity, adult adiposity, and cardiovascular risk factors. N Engl J Med. (2011) 365:1876–85. doi: 10.1056/nejmoa1010112, PMID: 22087679

[11] Du T Fonseca V Chen W Bazzano LA. Changes in body size phenotypes from childhood to adulthood and the associated cardiometabolic outcomes. Diabetes Res Clin Pract. (2022) 187:109884. doi: 10.1016/j.diabres.2022.109884, PMID: 35487340

[12] Jacob M Lopata AL Dasouki M Abdel Rahman AM. Metabolomics toward personalized medicine. Mass Spectrom Rev. (2019) 38:221–38. doi: 10.1002/mas.21548, PMID: 29073341

[13] Zhang A Sun H Wang X. Power of metabolomics in biomarker discovery and mining mechanisms of obesity. Obes Rev. (2013) 14:344–9. doi: 10.1111/obr.12011, PMID: 23279162

[14] Xie G Wang L Chen T Zhou K Zhang Z Li J. A metabolite array technology for precision medicine. Anal Chem. (2021) 93:5709–17. doi: 10.1021/acs.analchem.0c04686, PMID: 33797874

[15] Lin C Tian Q Guo S Xie D Cai Y Wang Z. Metabolomics for clinical biomarker discovery and therapeutic target identification. Molecules. (2024) 29:2198. doi: 10.3390/molecules29102198, PMID: 38792060 PMC11124072

[16] Soria-Gondek A Fernández-García P González L Reyes-Farias M Murillo M Valls A. Lipidome profiling in childhood obesity compared to adults: A pilot study. Nutrients. (2023) 15:3341. doi: 10.3390/nu15153341, PMID: 37571279 PMC10421258

[17] Szczerbinski L Wojciechowska G Olichwier A Taylor MA Puchta U Konopka P. Untargeted metabolomics analysis of the serum metabolic signature of childhood obesity. Nutrients. (2022) 14:214. doi: 10.3390/nu14010214, PMID: 35011090 PMC8747180

[18] Perng W Gillman MW Fleisch AF Michalek RD Watkins SM Isganaitis E. Metabolomic profiles and childhood obesity. Obes (Silver Spring). (2014) 22:2570–8. doi: 10.1002/oby.20901, PMID: 25251340 PMC4236243

[19] Su Z Zeng C Huang J Luo S Guo J Fu J. Association of dietary patterns, C-reactive protein, and risk of obesity among children aged 9–17 years in Guangzhou, China: A cross-sectional mediation study. Nutrients. (2024) 16:3835. doi: 10.3390/nu16223835, PMID: 39599620 PMC11597664

[20] National Health and Family Planning Commission of the People’s Republic of China. Screen for Overweight and Obesity among School-Age Children and Adolescents (2018). Available online at: http://www.nhc.gov.cn/wjw/pqt/201803/a7962d1ac01647b9837110bfd2d69b26.shtml (Accessed March 15, 2025).

[21] Damanhoury S Newton AS Rashid M Hartling L Byrne JLS Ball GDC. Defining metabolically healthy obesity in children: a scoping review. Obes Rev. (2018) 19:1476–91. doi: 10.1111/obr.12721, PMID: 30156016

[22] Vukovic R Santos TJD Ybarra M Atar M. Children with metabolically healthy obesity: A review. Front Endocrinol. (2019) 10:865. doi: 10.3389/fendo.2019.00865, PMID: 31920976 PMC6914809

[23] Dong Y Ma J Song Y Dong B Wang Z Yang Z. National blood pressure reference for chinese han children and adolescents aged 7 to 17 years. Hypertension. (2017) 70:897–906. doi: 10.1161/HYPERTENSIONAHA.117.09983, PMID: 28923902 PMC5722224

[24] Ministry of Health of the People’s Republic of China. Technical Standard for Physical Examination for Students:GB/T 26343-2010 (2011). Available online at: http://www.nhc.gov.cn/wjw/pqt/201106/51939.shtml (Accessed May 20, 2025).

[25] Chen P Wang D Shen H Yu L Gao Q Mao L. Physical activity and health in Chinese children and adolescents: expert consensus statement (2020). Br J Sports Med. (2020) 54:1321–31. doi: 10.1136/bjsports-2020-102261, PMID: 32471813 PMC7606574

[26] Zheng G Xia H Lai Z Shi H Zhang J Wang C. Dietary inflammatory index and dietary diversity score associated with sarcopenia and its components: findings from a nationwide cross-sectional study. Nutrients. (2024) 16:1038. doi: 10.3390/nu16071038, PMID: 38613070 PMC11013103

[27] Liu X Liu C Chen K. Agricultural production diversity, child dietary diversity and nutritional status in poor, rural Gansu Province of China. PLoS One. (2023) 18:e0287000. doi: 10.1371/journal.pone.0287000, PMID: 37315059 PMC10266675

[28] Kroese LJ Scheffer PG. 8-hydroxy-2′-deoxyguanosine and cardiovascular disease: a systematic review. Curr Atheroscler Rep. (2014) 16:452. doi: 10.1007/s11883-014-0452-y, PMID: 25252787

[29] Hahm JY Park J Jang E-S Chi SW. 8-Oxoguanine: from oxidative damage to epigenetic and epitranscriptional modification. Exp Mol Med. (2022) 54:1626–42. doi: 10.1038/s12276-022-00822-z, PMID: 36266447 PMC9636213

[30] Di Minno A Turnu L Porro B Squellerio I Cavalca V Tremoli E. 8-hydroxy-2-deoxyguanosine levels and cardiovascular disease: A systematic review and meta-analysis of the literature. Antioxid Redox Signal. (2016) 24:548–55. doi: 10.1089/ars.2015.6508, PMID: 26650622 PMC4827317

[31] Ishiguchi H Kobayashi S Myoren T Kohno M Nanno T Murakami W. Urinary 8-hydroxy-2’-deoxyguanosine as a myocardial oxidative stress marker is associated with ventricular tachycardia in patients with active cardiac sarcoidosis. Circ Cardiovasc Imaging. (2017) 10:e006764. doi: 10.1161/circimaging.117.006764, PMID: 29208596

[32] Baslow MH. A review of phylogenetic and metabolic relationships between the acylamino acids, N-acetyl-L-aspartic acid and N-acetyl-L-histidine, in the vertebrate nervous system. J Neurochem. (1997) 68:1335–44. doi: 10.1046/j.1471-4159.1997.68041335.x, PMID: 9084403

[33] Yao X Yang C Jia X Yu Z Wang C Zhao J. High-fat diet consumption promotes adolescent neurobehavioral abnormalities and hippocampal structural alterations via microglial overactivation accompanied by an elevated serum free fatty acid concentration. Brain Behav Immun. (2024) 119:236–50. doi: 10.1016/j.bbi.2024.04.005, PMID: 38604269

[34] Björntorp P Rosmond R. Obesity and cortisol. Nutrition. (2000) 16:924–36. doi: 10.1016/s0899-9007(00)00422-6, PMID: 11054598

[35] Dammann C Stapelfeld C Maser E. Expression and activity of the cortisol-activating enzyme 11β-hydroxysteroid dehydrogenase type 1 is tissue and species-specific. Chemico-Biological Interact. (2019) 303:57–61. doi: 10.1016/j.cbi.2019.02.018, PMID: 30796905

[36] Hughes KA Manolopoulos KN Iqbal J Cruden NL Stimson RH Reynolds RM. Recycling between cortisol and cortisone in human splanchnic, subcutaneous adipose, and skeletal muscle tissues. vivo. Diabetes. (2012) 61:1357–64. doi: 10.2337/db11-1345, PMID: 22511204 PMC3357308

[37] Pereira CD Azevedo I Monteiro R Martins MJ. 11β-Hydroxysteroid dehydrogenase type 1: relevance of its modulation in the pathophysiology of obesity, the metabolic syndrome and type 2 diabetes mellitus. Diabetes Obes Metab. (2012) 14:869–81. doi: 10.1111/j.1463-1326.2012.01582.x, PMID: 22321826

[38] Anderson AJ Andrew R Homer NZM Hughes KA Boyle LD Nixon M. Effects of obesity and insulin on tissue-specific recycling between cortisol and cortisone in men. J Clin Endocrinol Metab. (2021) 106:e1206–20. doi: 10.1210/clinem/dgaa896, PMID: 33270115 PMC7947841

[39] Purnell JQ Kahn SE Samuels MH Brandon D Loriaux DL Brunzell JD. Enhanced cortisol production rates, free cortisol, and 11beta-HSD-1 expression correlate with visceral fat and insulin resistance in men: effect of weight loss. Am J Physiol Endocrinol Metab. (2009) 296:E351–357. doi: 10.1152/ajpendo.90769.2008, PMID: 19050176 PMC2645022

[40] Nijm J Jonasson L. Inflammation and cortisol response in coronary artery disease. Ann Med. (2009) 41:224–33. doi: 10.1080/07853890802508934, PMID: 18979272

[41] Chapman KE Coutinho A Gray M Gilmour JS Savill JS Seckl JR. Local amplification of glucocorticoids by 11β-hydroxysteroid dehydrogenase type 1 and its role in the inflammatory response. Ann New York Acad Sci. (2006) 1088:265–73. doi: 10.1196/annals.1366.030, PMID: 17192572

[42] Cooper MS Stewart PM. 11Beta-hydroxysteroid dehydrogenase type 1 and its role in the hypothalamus-pituitary-adrenal axis, metabolic syndrome, and inflammation. J Clin Endocrinol Metab. (2009) 94:4645–54. doi: 10.1210/jc.2009-1412, PMID: 19837912

[43] Jedynak P Bustamante M Rolland M Mustieles V Thomsen C Sakhi AK. Prenatal exposure to synthetic phenols assessed in multiple urine samples and dysregulation of steroid hormone homeostasis in two european cohorts. Environ Health Perspect. (2025) 133:57011. doi: 10.1289/ehp15117, PMID: 40117576 PMC12097533

[44] Li M Chang Q Luo Y Pan J Hu Y Liu B. The gut microbial composition in polycystic ovary syndrome with hyperandrogenemia and its association with steroid hormones. Front Cell Dev Biol. (2024) 12:1384233. doi: 10.3389/fcell.2024.1384233, PMID: 38872933 PMC11169812

[45] Guillet C Masgrau A Boirie Y. Is protein metabolism changed with obesity? Curr Opin Clin Nutr Metab Care. (2011) 14:89–92. doi: 10.1097/mco.0b013e328341389e, PMID: 21088567

[46] Bergen WG. Protein synthesis in animal models. J Anim Sci. (1974) 38:1079–91. doi: 10.2527/jas1974.3851079x, PMID: 4601493

[47] Xue X Piao J-H Nakajima A Sakon-Komazawa S Kojima Y Mori K. Tumor necrosis factor alpha (TNFalpha) induces the unfolded protein response (UPR) in a reactive oxygen species (ROS)-dependent fashion, and the UPR counteracts ROS accumulation by TNFalpha. J Biol Chem. (2005) 280:33917–25. doi: 10.1074/jbc.m505818200, PMID: 16107336

[48] Guillet C Masgrau A Walrand S Boirie Y. Impaired protein metabolism: interlinks between obesity, insulin resistance and inflammation. Obes Rev. (2012) 13:51–7. doi: 10.1111/j.1467-789x.2012.01037.x, PMID: 23107259

[49] Henein MY Vancheri S Longo G Vancheri F. The role of inflammation in cardiovascular disease. Int J Mol Sci. (2022) 23:12906. doi: 10.3390/ijms232112906, PMID: 36361701 PMC9658900

[50] Mouton AJ Li X Hall ME Hall JE. Obesity, hypertension, and cardiac dysfunction: novel roles of immunometabolism in macrophage activation and inflammation. Circ Res. (2020) 126:789–806. doi: 10.1161/circresaha.119.312321, PMID: 32163341 PMC7255054

[51] Evans JL Goldfine ID Maddux BA Grodsky GM. Are oxidative stress-activated signaling pathways mediators of insulin resistance and beta-cell dysfunction? Diabetes. (2003) 52:1–8. doi: 10.2337/diabetes.52.1.1, PMID: 12502486

[52] Guzik TJ Touyz RM. Oxidative stress, inflammation, and vascular aging in hypertension. Hypertension. (2017) 70:660–7. doi: 10.1161/hypertensionaha.117.07802, PMID: 28784646

[53] Xiao L Harrison DG. Inflammation in hypertension. Can J Cardiol. (2020) 36:635–47. doi: 10.1016/j.cjca.2020.01.013, PMID: 32389337 PMC7733126

[54] Qi J Lv Y Zhong N-E Han W-Q Gou Q-L Sun C-F. Multi-omics analysis identifies potential mechanisms by which high glucose accelerates macrophage foaming. Mol Cell Biochem. (2023) 478:665–78. doi: 10.1007/s11010-022-04542-w, PMID: 36029453

[55] Shimizu K Ono M Mikamoto T Urayama Y Yoshida S Hase T. Overexpression of lysophospholipid acyltransferase, LPLAT10/LPCAT4/LPEAT2, in the mouse liver increases glucose-stimulated insulin secretion. FASEB J. (2024) 38:e23425. doi: 10.1096/fj.202301594rr, PMID: 38226852

[56] Cole LK Vance JE Vance DE. Phosphatidylcholine biosynthesis and lipoprotein metabolism. Biochim Biophys Acta. (2012) 1821:754–61. doi: 10.1016/j.bbalip.2011.09.009, PMID: 21979151

[57] Bagheri S Zolghadri S Stanek A. Beneficial effects of anti-inflammatory diet in modulating gut microbiota and controlling obesity. Nutrients. (2022) 14:3985. doi: 10.3390/nu14193985, PMID: 36235638 PMC9572805

[58] López-Gil JF García-Hermoso A Sotos-Prieto M Cavero-Redondo I Martínez-Vizcaíno V Kales SN. Mediterranean diet-based interventions to improve anthropometric and obesity indicators in children and adolescents: A systematic review with meta-analysis of randomized controlled trials. Adv Nutr. (2023) 14:858–69. doi: 10.1016/j.advnut.2023.04.011, PMID: 37127186 PMC10334150

[59] Razquin C Martinez-Gonzalez MA. A traditional mediterranean diet effectively reduces inflammation and improves cardiovascular health. Nutrients. (2019) 11:1842. doi: 10.3390/nu11081842, PMID: 31395816 PMC6723673

[60] Asemi Z Samimi M Tabassi Z Sabihi S Esmaillzadeh A. A randomized controlled clinical trial investigating the effect of DASH diet on insulin resistance, inflammation, and oxidative stress in gestational diabetes. Nutrition. (2013) 29:619–24. doi: 10.1016/j.nut.2012.11.020, PMID: 23466048

[61] Couch SC Saelens BE Khoury PR Dart KB Hinn K Mitsnefes MM. Dietary approaches to stop hypertension dietary intervention improves blood pressure and vascular health in youth with elevated blood pressure. Hypertension. (2021) 77:241–51. doi: 10.1161/hypertensionaha.120.16156, PMID: 33190559 PMC7725858

[62] Oakes EG Vlasakov I Kotler G Bubes V Mora S Tatituri R. Joint effects of one year of marine omega-3 fatty acid supplementation and participant dietary fish intake upon circulating lipid mediators of inflammation resolution in a randomized controlled trial. Nutrition. (2024) 123:112413. doi: 10.1016/j.nut.2024.112413, PMID: 38518540 PMC11088505

[63] Jin Q Liu T Qiao Y Liu D Yang L Mao H. Oxidative stress and inflammation in diabetic nephropathy: role of polyphenols. Front Immunol. (2023) 14:1185317. doi: 10.3389/fimmu.2023.1185317, PMID: 37545494 PMC10401049

[64] Maleki SJ Crespo JF Cabanillas B. Anti-inflammatory effects of flavonoids. Food Chem. (2019) 299:125124. doi: 10.1016/j.foodchem.2019.125124, PMID: 31288163

[65] Vinga S. Structured sparsity regularization for analyzing high-dimensional omics data. Brief Bioinform. (2021) 22:77–87. doi: 10.1093/bib/bbaa122, PMID: 32597465

[66] Anwardeen NR Diboun I Mokrab Y Althani AA Elrayess MA. Statistical methods and resources for biomarker discovery using metabolomics. BMC Bioinf. (2023) 24:250. doi: 10.1186/s12859-023-05383-0, PMID: 37322419 PMC10266963

[67] Bagheri M Farzadfar F Qi L Yekaninejad MS Chamari M Zeleznik OA. Obesity-related metabolomic profiles and discrimination of metabolically unhealthy obesity. J Proteome Res. (2018) 17:1452–62. doi: 10.1021/acs.jproteome.7b00802, PMID: 29493238

