# Supplementary material for: Obesity metabolomics signature in children: associations with metabolic abnormalities and potential biomarkers
Source: Front Endocrinol (Lausanne). 2025 Sep 25;16:1671613. doi: 10.3389/fendo.2025.1671613 (PMC12507617; doi:10.3389/fendo.2025.1671613)
Supplement: Supplementary file 1 [file DataSheet1.pdf]

Supplementary Material

1 LC-MS analysis process

1.1 Sample preparation and extraction

After collected from participants, fasting venous blood samples stored at -80℃ refrigerator were thawed on ice and vortexed for 10s. 50 μ L of each sample and 300 μ L of extraction solution, which included internal standards and had an ACN to Methanol ratio of 1:4 (v/v), were added into a 2mL microcentrifuge tube and vortexed for 3min. After centrifugation(4℃, 10min, 12000rpm), 200 μ L of the supernatant was collected and placed in -20℃ for 30min, and then centrifuged at 12000 rpm for 3min (4℃). A 180 μ L aliquots of supernatant were transferred for LC-MS analysis.

1.2 T3 UPLC Conditions

The sample extracts were analyzed using an LC-ESI-MS/MS system (UPLC, ExionLC AD, https://sciex.com.cn/; MS, QTRAP® System, https://sciex.com/). Chromatographic separation was performed on the Waters ACQUITY UPLC HSS T3 C18 column(1.8µm, 2.1mm×100mm) with the mobile phases A of 0.1% formic acid in water and B of 0.1% formic acid in acetonitrile at column temperature of 40°C and a flow rate of 0.4mL/min. Gradient elution program was as follows: increased from 5% to 20% B in 2min, increased from 20% to 60% B in the following 3min, increased to 99% B in 1min and held at 99% B for 1.5min, and then decreased from 99% to 5% B within 0.1min and maintained for 2.4 min. The injection volume was 2µL.

1.3 ESI-QTRAP-MS/MS

After separation by UPLC, mass spectrometry was conducted using a triple quadrupole-linear ion trap (QTRAP), equipped with an electrospray ionization ESI Turbo Ion-Spray interface (AB Sciex). LIT and triple quadrupole (QQQ) scans were acquired on a triple quadrupole-linear ion trap mass spectrometer (QTRAP). Measurements were conducted using the QTRAP®6500+ LC-MS/MS system, equipped with an ESI Turbo Ion-Spray interface, operating in both positive and negative ion modes, and controlled by Analyst 1.6.3 software (AB Sciex).

The ESI source operation parameters were as follows: source temperature 550°C; ion spray voltage (IS) 5500V under positive ion mode (or -4500V in negative ion mode); ion source gas I (GSI) 55 psi, gas II (GSII) 60 psi, curtain gas (CUR) 25 psi respectively; the collision gas (CAD) high. Instrument tuning and mass calibration were performed with 10 and 100µmol/L polypropylene glycol solutions in QQQ and LIT modes, respectively. Specific MRM transitions were monitored for each period based on the eluted compounds.

1.4 Metabolites and lipids qualitative and quantitative analysis

Qualitative analysis of the detected precursor ion and fragments spectra was conducted on the basis of self-built metware database (MWDB) with retention time and ion pairs, combined with the public database of metabolites information.

Quantification analysis was carried out by the multiple reaction monitoring (MRM) mode of a triple quadrupole mass spectrometer. Under the MRM mode, the quadrupole rod first selected the precursor ions (parent ions) of the target substance, excluding ions corresponding to other molecular weight substances to initially eliminate interference. The precursor ions, after ionizing and breaking down in the collision cell, formed numerous fragment ions. These fragment ions were subsequently filtered through the triple quadrupole to select a characteristic fragment ion, thereby excluding non-target ions and enhancing the accuracy and repeatability of quantification. Following the acquisition of metabolite spectral data from various samples, integration of peak areas was conducted for the mass spectrum peaks, and corrections were applied to the peak areas of the same metabolite across different samples.

1.5 Quality control

Quality control (QC) samples are prepared by mixing extracts of the samples to assess the repeatability of the analysis under the same treatment conditions. During the instrumental analysis, typically one QC sample is inserted every 10 analytical samples to monitor the reproducibility of the analysis process. By overlapping and analyzing the total ion current (TIC) chromatograms from different QC samples, the repeatability of metabolite extraction and detection can be evaluated, which refers to the technical repeatability. The high stability of the instrument provides an essential guarantee for the repeatability and reliability of the data (Supplementary Figure 1).

(A) 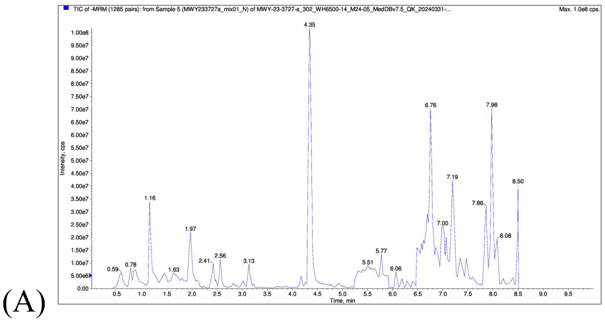

(B) 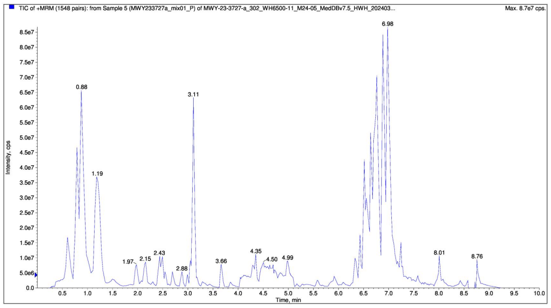

Supplementary Figure 1. Mixed sample quality control mass spectrometry total ion current (TIC) diagram. (A) and (B) showed metabolic profiling under positive ion mode and negative ion mode, respectively.

2 Supplementary figures

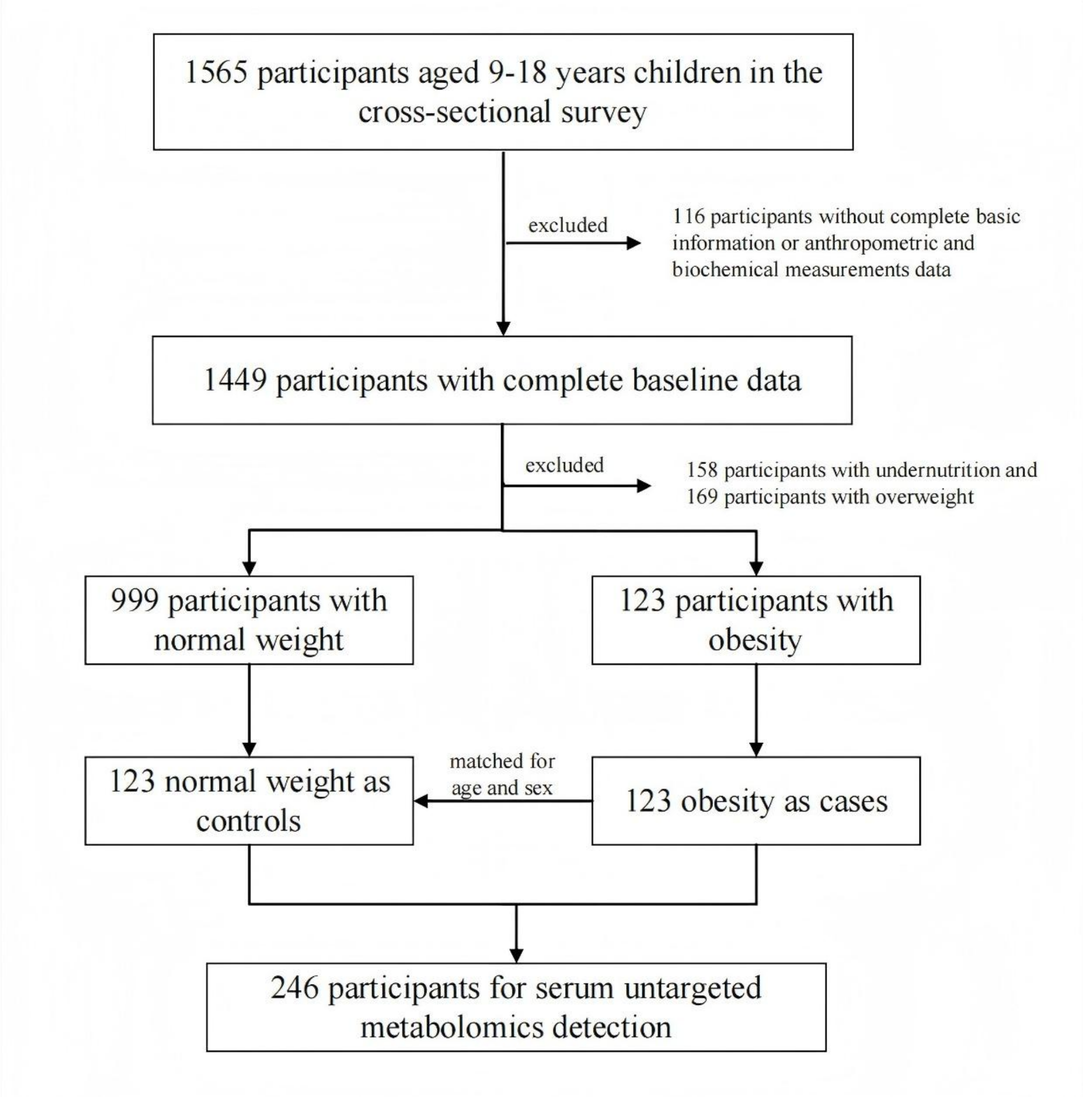

Supplementary Figure 2. Flowchart of the selection of research participants.

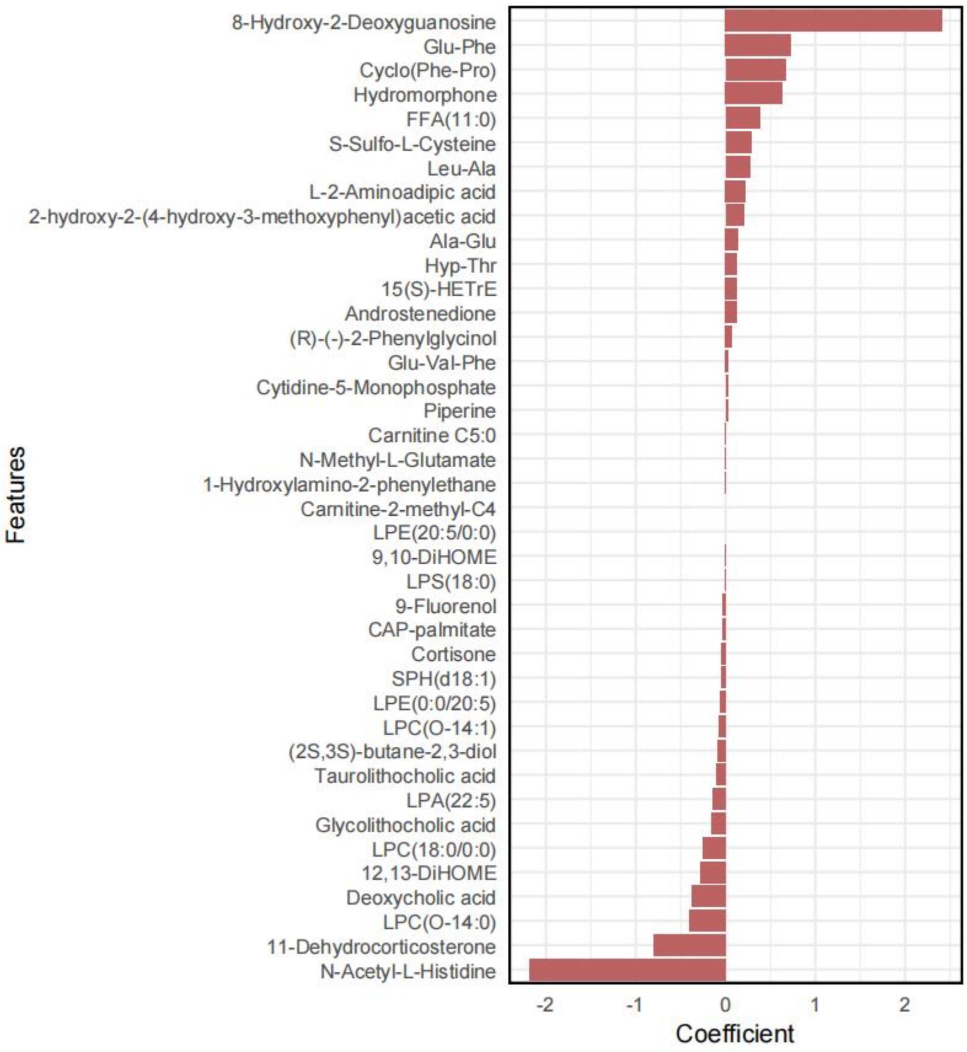

Supplementary Figure 3. Regression coefficients of obesity-associated candidate metabolites selected by LASSO analysis

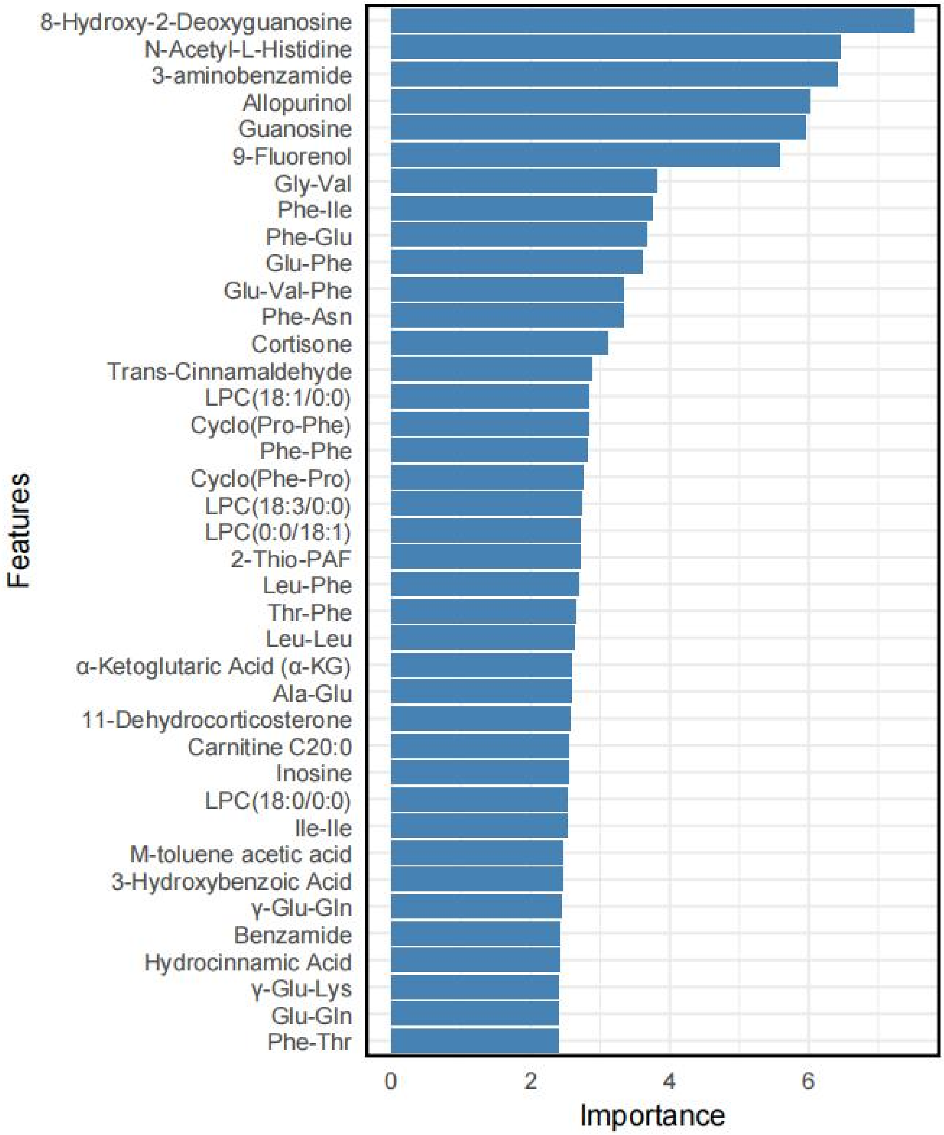

Supplementary Figure 4. Importance ranking of obesity-associated candidate metabolites selected by RF-RFE

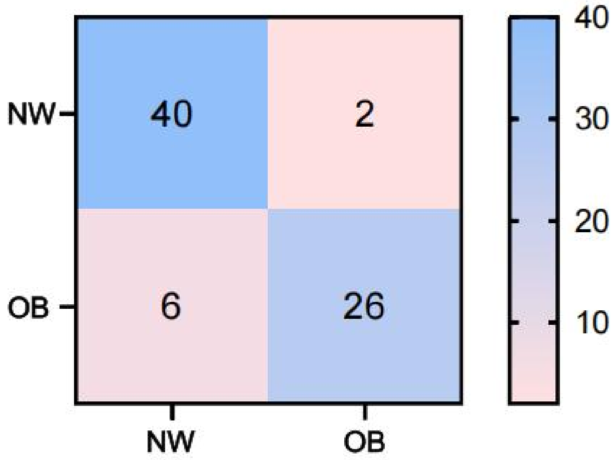

Supplementary Figure 5. The confusion matrix of the testing set (n=74) based on final logistic model
